# Malignancy risk stratification for pulmonary nodules: comparing a deep learning approach to multiparametric statistical models in different disease groups

**DOI:** 10.1007/s00330-024-11256-8

**Published:** 2025-01-02

**Authors:** Lars Piskorski, Manuel Debic, Oyunbileg von Stackelberg, Kai Schlamp, Linn Welzel, Oliver Weinheimer, Alan Arthur Peters, Mark Oliver Wielpütz, Thomas Frauenfelder, Hans-Ulrich Kauczor, Claus Peter Heußel, Jonas Kroschke

**Affiliations:** 1 Diagnostic and Interventional Radiology, Heidelberg University Hospital, Heidelberg, Germany; 2 Translational Lung Research Center Heidelberg (TLRC), Member of the German Center for Lung Research (DZL), Heidelberg, Germany; 3 Diagnostic and Interventional Radiology with Nuclear Medicine, Thoraxklinik, Heidelberg University Hospital, Heidelberg, Germany; 4 Department for Diagnostic, Interventional and Pediatric Radiology, Inselspital, Bern University Hospital, University of Bern, Bern, Switzerland; 5 Diagnostic and Interventional Radiology, University Hospital Zurich, Zurich, Switzerland

**Keywords:** Lung neoplasm, Risk assessment, Decision support, Emphysema, Interstitial lung disease

## Abstract

**Objectives:**

Incidentally detected pulmonary nodules present a challenge in clinical routine with demand for reliable support systems for risk classification. We aimed to evaluate the performance of the lung-cancer-prediction-convolutional-neural-network (LCP-CNN), a deep learning-based approach, in comparison to multiparametric statistical methods (Brock model and Lung-RADS®) for risk classification of nodules in cohorts with different risk profiles and underlying pulmonary diseases.

**Materials and methods:**

Retrospective analysis was conducted on non-contrast and contrast-enhanced CT scans containing pulmonary nodules measuring 5–30 mm. Ground truth was defined by histology or follow-up stability. The final analysis was performed on 297 patients with 422 eligible nodules, of which 105 nodules were malignant. Classification performance of the LCP-CNN, Brock model, and Lung-RADS® was evaluated in terms of diagnostic accuracy measurements including ROC-analysis for different subcohorts (total, screening, emphysema, and interstitial lung disease).

**Results:**

LCP-CNN demonstrated superior performance compared to the Brock model in total and screening cohorts (AUC 0.92 (95% CI: 0.89–0.94) and 0.93 (95% CI: 0.89–0.96)). Superior sensitivity of LCP-CNN was demonstrated compared to the Brock model and Lung-RADS® in total, screening, and emphysema cohorts for a risk threshold of 5%. Superior sensitivity of LCP-CNN was also shown across all disease groups compared to the Brock model at a threshold of 65%, compared to Lung-RADS® sensitivity was better or equal. No significant differences in the performance of LCP-CNN were found between subcohorts.

**Conclusion:**

This study offers further evidence of the potential to integrate deep learning-based decision support systems into pulmonary nodule classification workflows, irrespective of the individual patient risk profile and underlying pulmonary disease.

**Key Points:**

***Question*** *Is a deep-learning approach (LCP-CNN) superior to multiparametric models (Brock model, Lung-RADS*®*) in classifying pulmonary nodule risk across varied patient profiles*?

***Findings*** *LCP-CNN shows superior performance in risk classification of pulmonary nodules compared to multiparametric models with no significant impact on risk profiles and structural pulmonary diseases*.

***Clinical relevance*** *LCP-CNN offers efficiency and accuracy, addressing limitations of traditional models, such as variations in manual measurements or lack of patient data, while producing robust results. Such approaches may therefore impact clinical work by complementing or even replacing current approaches*.

## Introduction

Pulmonary nodules are a common finding in chest computed tomography (CT) scans that pose an ongoing management problem when detected, as their etiology can range from benign findings to lung cancer, which often cannot be differentiated by visual inspection alone. The rising number of chest CT scans, exacerbated by the increasing implementation of lung cancer screening programs, is placing an increasing workload on radiologists as more nodules are detected. In the US alone, approximately 1.56 million pulmonary nodules are diagnosed each year with incidental nodules found in nearly 31% of all chest CT examinations [1]. Effective risk stratification of lung nodules is therefore needed in clinical practice to avoid unnecessary follow-up, invasive procedures to characterize nodules, and uncertainty for patients if misclassified as malignant and delay or lack of treatment if misclassified as benign.

In clinical practice, probably the most widely used system for the management of pulmonary nodules are the guidelines published by the Fleischner Society [2]. These guidelines are based on the number of nodules and their average nodule diameter or volume while stratifying patients into low- and high-risk groups. Resulting recommendations range from no need for follow-up for low-risk patients with small nodules to positron emission tomography/CT or tissue sampling for high-risk patients with large nodules. However, these guidelines are only validated for patients outside of the lung cancer screening population, as those have a different risk profile for lung cancer.

The Brock University model is a multivariable statistical approach developed specifically for the screening setting in the context of the Pan-Canadian Early Detection of Lung Cancer Study [3–6]. This model combines nodule characteristics and patient characteristics to calculate the risk of malignancy for lung nodules. Management guidelines are not proposed by this model. Although developed for the lung cancer screening setting, the Brock model has also been validated in patient cohorts with incidental pulmonary nodules [7, 8]. However, in many clinical situations, radiologists have limited information about patient characteristics, which reduces the accuracy of the model.

The American College of Radiology has proposed a system for the management of pulmonary nodules detected in the context of lung cancer screening: Lung Imaging Reporting and Data System (Lung-RADS^®^) [9, 10], currently in its third version from 2022. Like Fleischner guidelines, Lung-RADS^®^ focuses on the imaging assessment of pulmonary nodules, taking into account nodule size, morphology, and location, classifying nodules into one of six groups with a corresponding risk of malignancy ranging from < 1% for scores 1–2 to > 15% in the highest score groups (4B and 4X). Resulting management recommendations range from yearly screening continuation to further imaging workup or tissue sampling. Although strictly not developed for incidental pulmonary nodules, the similarities of Lung-RADS^®^ and Fleischner guidelines, along with its widespread use in screening settings, make it an interesting risk stratification model for further investigation in patient cohorts with incidental pulmonary nodules.

Although established methods for risk stratification of pulmonary nodules exist, there are still limitations. These methods can be time-consuming, requiring careful evaluation of nodule characteristics or even the need to include patient characteristics that might not be readily available when reading a study.

The integration of artificial intelligence into radiology workflows has the potential to substantially alter the way radiologists work, with the objective of reducing workload and possibly providing support in decision-making [11, 12]. The company Optellum has developed a deep learning-based approach for malignancy prediction of pulmonary nodules, the LCP-CNN score. The model was trained on the dataset from the National Lung Cancer Screening Trial [13, 14] and subsequently validated in the LUCINDA study (early lung cancer diagnosis using artificial intelligence and big data) [15]. Different authors have investigated the performance of the LCP-CNN score, demonstrating better performance compared to the Brock model [16] and the Mayo Clinic model [13], as well as examining the implications for nodule management compared to British Thoracic Society guidelines [17] or looking at the performance with changing imaging parameters [18]. The Food and Drug Administration (FDA)-approved and clinically available version used in this study classifies pulmonary nodules into ten categories with corresponding relative risk thresholds (see Table 1). A dataset was derived from clinical practice at Thoraxklinik, University Hospital Heidelberg, including patients with pulmonary nodules. The study employed minimal exclusion criteria to better reflect everyday clinical practice. Based on the ground truth the AI-based Optellum LCP-CNN score was then compared to the Brock model and Lung-RADS^®^, with the addition of subclassifying patients with underlying pulmonary structural changes due to emphysema or fibrosis and further evaluating possible effects on the investigated risk stratification model performance.

**Table 1:** Results of the risk assessment for all nodules with LCP-CNN, Brock model, and Lung-RADS^®^ (Lung-RADS^®^ category 1 is not included, as this category is used when no nodule is found)

	Nodule risk assessment results									
	Low risk				Intermediate risk			High risk		
LCP-CNN score (relative risk)	1 (0.2%)	2 (0.4%)	3 (0.8%)	4 (2%)	5 (5.6%)	6 (15%)	7 (34%)	8 (64%)	9 (84%)	10 (93%)
Benign (*n* = 317)	47 (15%)	47 (15%)	42 (13%)	39 (12%)	47 (15%)	38 (12%)	20 (6%)	24 (8%)	8 (3%)	5 (2%)
Malignant (*n* = 105)	0	0	0	3 (3%)	4 (4%)	6 (6%)	13 (12%)	21 (20%)	19 (18%)	39 (37%)
Total (*n* = 422)	47 (11%)	47 (11%)	42 (10%)	42 (10%)	51 (12%)	44 (10%)	33 (8%)	45 (11%)	27 (6%)	44 (10%)
Brock model	< 0.4%	< 0.8%	< 2%	< 5.6%	< 15%	< 34%	< 64%	< 84%	< 93%	> 93%
Benign (*n* = 317)	9 (3%)	26 (8%)	85 (27%)	94 (30%)	65 (21%)	30 (9%)	8 (3%)	0	0	0
Malignant (*n* = 105)	0	1 (1%)	2 (2%)	8 (8%)	20 (19%)	40 (38%)	32 (30%)	2 (2%)	0	0
Total (*n* = 422)	9 (2%)	27 (6%)	87 (26%)	102 (24%)	85 (20%)	70 (17%)	40 (9%)	2 (0.5%)	0	0
LungRADS© (relative risk)	2 (< 1%)		3 (1–2%)		4A (5–15%)			4B (> 15%)	4X (> 15%)	
Benign (*n* = 317)	118 (37%)		84 (26%)		58 (18%)			4 (1%)	53 (17%)	
Malignant (*n* = 105)	2 (2%)		7 (7%)		23 (22%)			6 (6%)	67 (64%)	
Total (*n* = 422)	120 (28%)		91 (22%)		81 (19%)			10 (2%)	120 (28%)	

## Materials and methods

Ethics approval was granted by the local ethics committee and the study was conducted in accordance with the principles of the Helsinki Declaration.

### Patient cohort and CT examination criteria

**Fig. 1:**
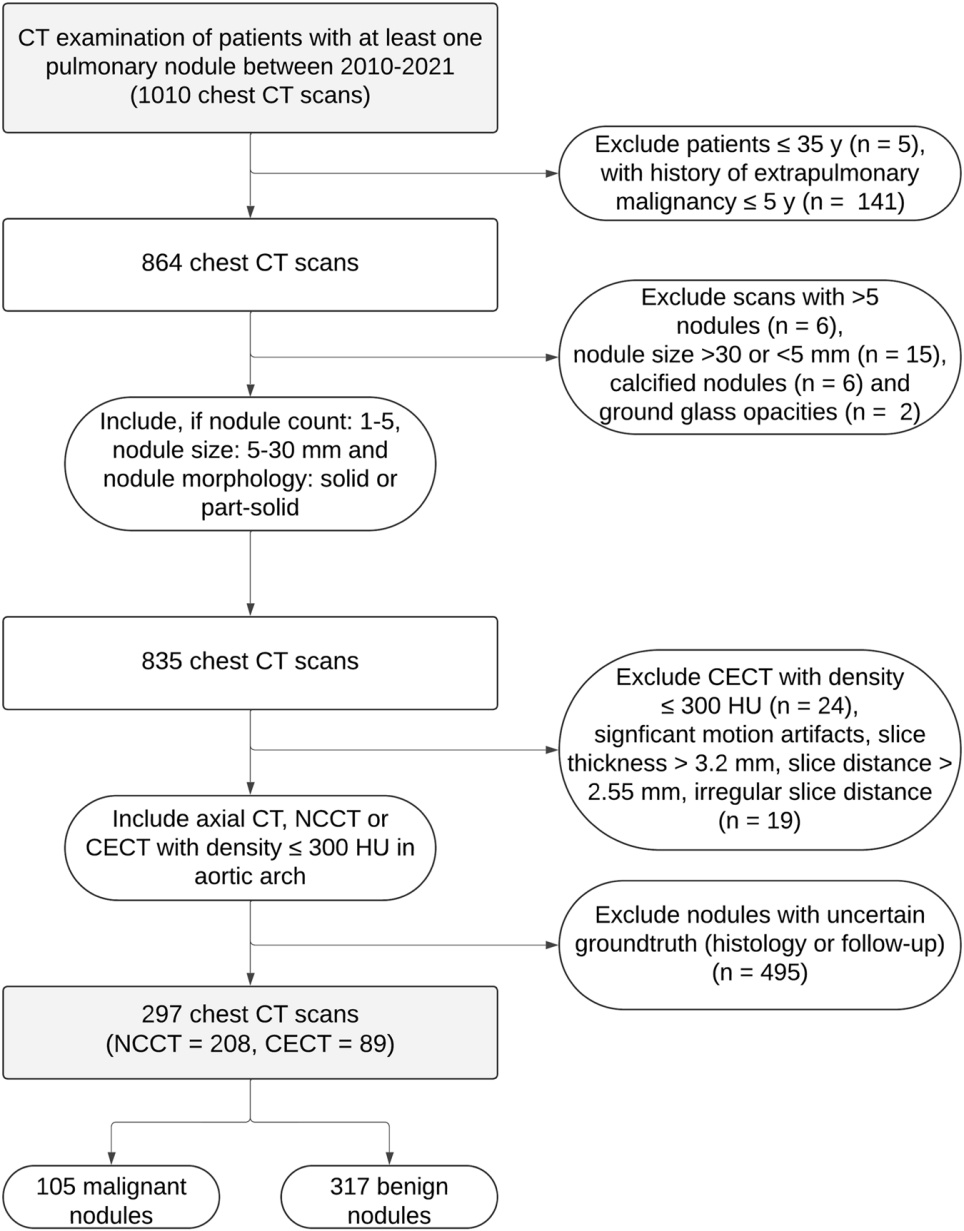
Patient flowchart

**Fig. 2:**
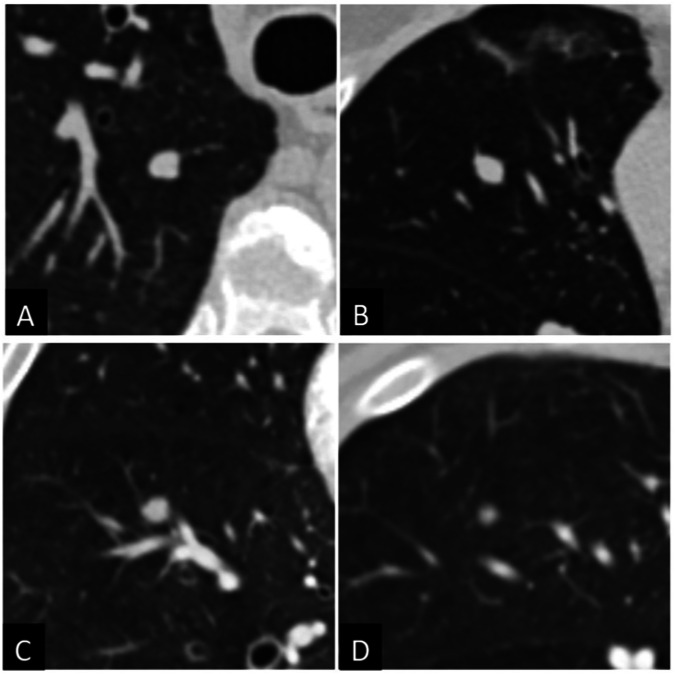
Examples of investigated incidental pulmonary nodules that were classified as false negatives by LCP-CNN (**A**, **B**) and Brock model and Lung-RADS^®^ (**C**, **D**)

Eligible studies were selected retrospectively from a patient population examined at Thoraxklinik at University Hospital Heidelberg, Germany, between 2010 and 2021. The cohort initially included patients found to have at least one pulmonary nodule, regardless of comorbidities, amounting to 1010 CT examinations of the chest in total. This represented the typical clinical case-mix at the institution. Criteria for inclusion and exclusion of patients/examinations are presented in Fig. 1 and were selected in accordance with the criteria defined by the FDA-approved version of the Optellum LCP-CNN score used in this study [19]. Only patients aged ≥ 35 years with no known history of extrapulmonary malignancy in the past 5 years were included. CT studies are required to include 1–5 pulmonary nodules, measuring 5–30 mm. Only solid or partly solid nodules were included with (partially) calcified nodules and ground-glass nodules being excluded. Examinations were included if they were performed without contrast or if the density in the aorta was measured below 300 Hounsfield Units in contrast-enhanced CTs with no beam hardening artifacts present. Nodule characteristics are summarized in Table 2. Figure 2 gives examples of investigated incidental pulmonary nodules that were classified as false negatives by LCP-CNN, Brock model, and Lung-RADS^®^. The study employed only the earliest available examination of each patient.

**Table 2:** Nodule characteristics

	Nodule characteristics		
	Total (*n* = 422)	Malignant (*n* = 105)	Benign (*n* = 317)
Diameter (mean, SD)	10.2 (4.6)	14.9 (4.8)	8.7 (3.3)
Size (*n*, %)			
5 mm	3 (0.7%)	0	3 (0.9%)
> 5–7 mm	122 (28.9%)	2 (1.9%)	120 (37.9%)
> 7–10 mm	128 (30.3%)	11 (10.5%)	117 (36.9%)
> 10–15 mm	108 (25.6%)	46 (43.8%)	62 (19.6%)
> 15–30 mm	61 (14.5%)	46 (43.8%)	15 (4.7%)
Localization (*n*, %)			
RUL	93 (22.0%)	36 (34.3%)	57 (18.0%)
ML	45 (10.7%)	8 (7.6%)	37 (11.7%)
RLL	108 (25.6%)	24 (22.9%)	84 (26.5%)
LUL	78 (18.5%)	27 (25.7%)	51 (16.1%)
LLL	98 (23.2%)	10 (9.5%)	88 (27.8%)
Perifissural (*n*, %)	29 (6.9%)	0	29 (9.1%)
Subpleural (*n*, %)	42 (10.0%)	0	42 (13.2%)
Morphology (*n*, %)			
Solid	399 (94.6%)	92 (87.6%)	307 (96.8%)
Part-solid	23 (5.5%)	13 (12.4%)	10 (3.2%)
Spiculation (*n*, %)	111 (26.3%)	63 (60.0%)	48 (15.1%)

All CT scans were performed on a 64-row CT scanner (Definition AS64, Siemens, Siemens Medical Solutions) in full inspiration supine position and with and without intravenous contrast administration. Standard acquisition parameters were used (100–120 kV and 70 mAs reference with dose modulation (Caredose 4D, Siemens), collimation 0.6 mm, re-constructed slice thickness 1.0 mm, and increment 0.8 mm in an iterative medium-soft kernel (I40f, SAFIRE level 3, Siemens)).

A total of 297 examinations met the defined criteria (208 non-contrast CT scans and 89 contrast-enhanced CT scans), containing 422 pulmonary nodules. Of these 105 were found to be malignant, while 317 nodules were benign findings. Malignancy was confirmed histologically, while benign nodules were defined either by histological results (mandatory for part-solid nodules), 2-year stability in nodule diameter, or 1-year stability in nodule volume.

Corresponding clinical patient data was extracted from the digital medical records archive. A summary of patient characteristics is presented in Table 3. Three subcohorts were derived from the total patient cohort: a screening cohort, an emphysema cohort, and an interstitial lung disease (ILD) cohort. The screening cohort comprised only patients meeting the criteria for inclusion into lung cancer screening as defined by the US Preventive Services Task Force (age between 50 years and 80 years, current smokers or ex-smokers within the last 15 years with at least 20 pack years of smoking history [20]). The emphysema cohort and ILD cohort comprise patients with a documented diagnosis of one of these pulmonary diseases made by specialists at the study site in a multidisciplinary approach. The study included some patients presenting with changes compatible with ILD in imaging, including for example usual interstitial pneumonia or non-specific interstitial pneumonia patterns as reported by a thoracic radiologist, but lacking a definitive diagnosis as defined by a multidisciplinary ILD board.

**Table 3:** Patient demographics and clinical characteristics

Patient demographics and characteristics Age, (years)			
	Total	Male patients	Female patients
Mean, SD	64.9 (9.5)	65.8 (9.5)	63.9 (9.3)
Median	65	66	64
IQR	59–72	60–73	57–71
	Total (*n* = 297)	Male patients (*n* = 167)	Female patients (*n* = 130)
Screening cohort (*n*, %)			
Screening	148 (49.8%)	83 (49.7%)	65 (50%)
Pulmonary disease (*n*, %)			
Emphysema	136 (45.8%)	74 (44.3%)	62 (47.7%)
ILD	47 (15.8%)	32 (19.2%)	15 (11.5%)
Smoking status (*n*, %)			
Current	47 (15.8%)	27 (16.2%)	20 (15.4%)
Former	180 (60.6%)	102 (61.1%)	78 (60.0%)
Never	39 (13.1%)	19 (11.4%)	20 (15.4%)
Unknown	31 (10.4%)	19 (11.4%)	12 (9.2%)
Family history of lung cancer (*n*, %)			
Negative	139 (46.8%)	74 (44.3%)	65 (50.0%)
Positive	22 (7.4%)	15 (9.0%)	7 (5.4%)
Unknown	136 (45.8%)	78 (46.7%)	58 (44.6%)

### Determining pulmonary nodule risk stratification scores

Pulmonary nodules were manually identified in CT examinations and their location (lung lobe), morphology, and size (mean diameter) were assessed. Nodule assessment was performed by a radiology resident (1 year of experience) under the supervision of an experienced chest radiologist (5 years of experience in chest radiology): In the beginning, a number of cases were assessed together, and then questionable cases were resolved by consensus.

Brock model was calculated by combining nodule characteristics (size, morphology, presence of spiculation, location, and number of nodules) and patient characteristics (age, sex, family history, and presence of pulmonary emphysema) using the “Nodule Malignancy Prediction Calculator (full model)” (version Tammemagi V1-2SEP13 [3]).

The Lung-RADS^®^ version 1.1 score was determined from nodule size, morphology, and location in accordance with the current guidelines proposed by the American College of Radiology [9].

The deep learning-based LCP-CNN score was derived by manually selecting the relevant nodule within the commercially available Optellum Nodule Lung Nodule Clinic, thus creating a region of interest (ROI) for software analysis.

### Statistical analysis

IBM SPSS Statistics software (version 27.0.0.0; IBM Corp.) and MedCalc software (version 20.115) were used for statistical analysis.

Receiver operating characteristics (ROC) curves were generated for two investigated prediction models (Brock Model and LCP-CNN), with the areas under the curve (AUC) and a 95% confidence interval (CI) calculated. The AUCs are compared using the DeLong test [21]. The level of significance is set at α = 0.05. ROC analysis was not performed for Lung-RADS^®^ as it is not a direct risk estimator with only six categorical outcome values. Ratio-scaled characteristics, namely mean, median, standard deviation (SD), and interquartile range were calculated. For nominal scaled characteristics, the frequency is reported as a percentage. Sensitivity, specificity, positive and negative diagnostic likelihood ratio (DLR), positive predictive value (PPV), negative predictive value (NPV), and accuracy for different cut-off values were calculated for the models and reported with the associated CI.

The Fleischner Society, in conjunction with the American College of Chest Physicians, has proposed classifying pulmonary nodules into three groups of individual risk for malignancy: high-risk, with a likelihood of malignancy of > 65%, low-risk, with a likelihood < 5% and intermediate-risk, with a likelihood in between [2]. For each of the three models, individual cut-off values were chosen that resemble the 5% (rule-out) and 65% (rule-in) threshold. Nodules with an LCP-CNN score of 1–4, a Lung-RADS^®^ of 1–3, and a Brock model of < 5% were classified as low-risk. Nodules with an LCP-CNN score of 8–10, a Lung-RADS^®^ score of 4B to 4X, and a Brock-Model Score of > 65% were classified as high-risk. Nodules with a value in between were classified as intermediate-risk nodules. According to these thresholds, the sensitivity, specificity, positive and negative DLRs, PPV, NPV, and accuracy are calculated and specified with the corresponding CI for all three models (LCP-CNN score, Brock model score, Lung-RADS^®^ score).

As this is an exploratory analysis, no formal sample size calculation was conducted. Consequently, all *p*-values are of a descriptive nature.

## Results

### Cohort and subcohorts

A total of 297 patients with a mean age of 64.9 years were included in this study (Table 3). One hundred thirty-six patients had a previous diagnosis of pulmonary emphysema, while 47 patients suffered from an ILD. The majority of patients were former smokers (*n* = 180, 60.6%), followed by current smokers (*n* = 47, 15.8%), and 39 patients had never smoked (13.1%). In 31 patients, the smoking status was not documented (10.4%). Family history for lung cancer was available for 161 patients, for 136 patients this information was not available, and a negative family history had to be assumed.

A total of 105 malignant and 317 benign pulmonary nodules were found (Table 2). A significant difference was observed in nodule size (*p* < 0.001) for malignant nodules with a mean [SD] average diameter of 14.9 mm [4.8] and benign nodules with a mean [SD] average diameter of 8.7 [3.3]. Most malignant nodules were identified in the upper pulmonary lobes (34.3% in the right upper lobe and 25.7% in the left upper lobe). No malignant nodules were observed subpleural or perifissural, which is consistent with cancer registration statistics and the literature [22, 23]. Spiculation was a much more common morphological feature in malignant nodules (60.0%) compared to benign nodules (15.1%).

### Descriptive statistics

Table 1 gives an overview of the performed risk assessment. For better comparison, Brock model results were grouped according to the risk categories defined for LCP-CNN. Lung-RADS^®^ results are sorted according to the relative risk attributed to each score [24]. For the Brock model, an average risk of malignancy of 11.8% (SD of 14.5%) was calculated with a minimum of 0.3% and a maximum of 79.6%.

Results of sensitivity, specificity, and DLRs, as well as positive and NPVs calculated for rule-in (5% threshold) and rule-out (65% threshold) for each model in every cohort, are shown in Table 4. No relevant differences in the performance of LCP-CNN could be found between the total cohort, screening cohort, and both pulmonary disease subgroups for emphysema and ILD. At a threshold value of 5%, the LCP-CNN score demonstrated superior sensitivity compared to the Brock model and Lung-RADS^®^ in the total, screening, and emphysema cohorts, with 97.1% in the total cohort compared to 91.4% in the Brock model and Lung-RADS^®^. In the ILD cohort, the LCP-CNN and Lung-RADS^®^ each demonstrated a sensitivity of 100%. Superior sensitivity of LCP-CNN was also shown at a threshold value of 65% across all disease groups compared to the Brock model, while compared to Lung-RADS^®^ sensitivity was better or equal. The specificity of LCP-CNN was found to be moderate and no superiority to the Brock model or Lung-RADS^®^ can be concluded. DLRs show strong diagnostic evidence for LCP-CNN across disease groups with no relevant differences between the groups. Furthermore, LCP-CNN demonstrated excellent NPVs in the total cohort and in the subgroups.

**Table 4:** Descriptive statistics of LCP-CNN, Brock model, and Lung-RADS^®^ in different patient cohorts for classification in high risk (65% threshold) and low risk (5% threshold)

	Threshold	Sensitivity (95% CI)	Specificity (95% CI)	DLR+ (95% CI)	DLR− (95% CI)	PPV (95% CI)	NPV (95% CI)
LCP-CNN							
Total	5%	97.1% (91.9%–99.4%)	55.2% (49.6%–60.8%)	2.17 (1.91–2.46)	0.05 (0.02–0.16)	41.8% (35.5%–48.3%)	98.3% (95.2%–99.7%)
	65%	75.2% (65.9%–83.1%)	88.3% (84.3%–91.7%)	6.45 (4.67–8.90)	0.28 (0.20–0.39)	68.1% (58.8%–76.5%)	91.5% (87.8%–94.4%)
Screening	5%	97.1% (90.1%–99.7%)	50.0% (41.3%–58.7%)	1.94 (1.63–2.31)	0.06 (0.01–0.23)	50.0% (41.3%–58.7%)	97.1% (90.1%–99.7%)
	65%	80.0% (68.7%–88.6%)	88.2% (81.6%–93.1%)	6.8 (4.23–10.93)	0.23 (0.14–0.36)	77.8% (66.4%–86.7%)	89.6% (83.1%–94.2%)
Emphysema	5%	95.7% (85.5%–99.5%)	54.2% (46.0%–62.2%)	2.09 (1.74–2.51)	0.08 (0.02–0.31)	38.8% (29.9%–48.3%)	97.7% (91.2%–99.7%)
	65%	76.6% (62.0%–87.7%)	85.8% (79.3%–90.9%)	5.4 (3.55–8.20)	0.27 (0.16–0.46)	62.1 (48.4%–74.5%)	92.4% (86.7%–96.1%)
ILD	5%	100% (63.1%–100%)	50.0% (38.0%–62.0%)	2 (1.59–2.52)	0	18.2% (8.2%–32.7%)	100% (90.3%–100%)
	65%	62.5% (24.5%–91.5%)	90.3% (81.0%–96.0%)	6.43 (2.65–15.58)	0.42 (0.17–1.02)	41.7% (15.2%–72.3%)	95.6% (87.6%–99.1%)
Brock							
Total	5%	91.4% (84.4%–96.0%)	64.4% (58.8%–69.6%)	2.56 (2.19–3.01)	0.13 (0.07–0.25)	45.9% (39.0%–52.9%)	95.8% (92.1%–98.1%)
	65%	0.95% (0.02%–5.2%)	100% (98.9%–100%)		0.99 (0.97–1.01)	100% (2.5%–100%)	75.3% (70.9%–79.4%)
Screening	5%	95.7% (88.0%–99.1%)	61.8% (53.1%–70.0%)	2.5 (2.01–3.12)	0.07 (0.02–0.21)	56.3% (46.9%–65.4%)	96.6% (90.3%–99.3%)
	65%	0 (0.0%–5.13%)	100% (97.3%–100%)		1 (1.00–1.00)		66.0% (59.1%–72.5%)
Emphysema	5%	93.6% (82.5%–98.7%)	64.5% (56.4%–72.0%)	2.64 (2.11–3.30)	0.10 (0.03–0.30)	44.4% (34.5%–54.8%)	97.1% (91.7%–99.4%)
	65%	2.1% (0.05%–11.3%)	100% (97.7%–100%)		0.98 (0.94–1.02)	100% (2.50%–100%)	77.1% (70.7%–82.7%)
ILD	5%	87.5% (47.4%–99.7%)	70.8% (58.9%–81.0%)	3 (1.92–4.68)	0.18 (0.03–1.11)	25.0% (10.7%–44.9%)	98.1% (89.7%–100%)
	65%	0% (0.0%–36.9%)	100% (95.0%–100.0%)		1 (1.00–1.00)		90% (81.2%–95.6%)
Lung-RADS©							
Total	5%	91.4% (84.4%–96.0%)	63.7% (58.2%–69.0%)	2.52 (2.15–2.95)	0.13 (0.07–0.25)	45.5% (38.7%–52.5%)	95.7% (92.1%–98.0%)
	65%	69.5% (59.8%–78.1%)	82.0% (77.3%–86.1%)	3.87 (2.96–5.05)	0.37 (0.28–0.50)	56.2% (47.2%–64.8%)	89.0% (84.9%–92.4%)
Screening	5%	92.9% (84.1%–97.6%)	62.5% (53.8%–70.7%)	2.48 (1.97–3.11)	0.11 (0.05–0.27)	56.0% (46.5%–65.2%)	94.4% (87.5%–98.2%)
	65%	81.4% (70.3%–89.7%)	77.2% (69.2%–84.0%)	3.57 (2.57–4.96)	0.24 (0.15–0.40)	64.8% (53.9%–74.7%)	89.0% (81.9%–94.0%)
Emphysema	5%	89.4% (76.9%–96.5%)	65.8% (57.8%–73.2%)	2.61 (2.06–3.32)	0.16 (0.07–0.37)	44.2% (34.0%–54.8%)	95.3% (89.4%–98.5%)
	65%	80.9% (66.7%–90.9%)	76.8% (69.3%–83.2%)	3.48 (2.53–4.79)	0.25 (0.14–0.45)	51.4% (39.4%–63.2%)	93.0% (87.1%–96.7%)
ILD	5%	100% (63.1%–100%)	68.1% (56.0%–78.6%)	3.13 (2.23–4.39)	0	25.8% (11.9%–44.6%)	100% (92.8%–100%)
	65%	62.5% (24.5%–91.5%)	80.6% (69.5%–88.9%)	3.21 (1.57–6.56)	0.47 (0.19–1.15)	26.3% (9.2%–51.2%)	95.1% (86.3%–99.0%)

### LCP-CNN outperforms the Brock model in ROC analysis in selected cohorts

**Fig. 3:**
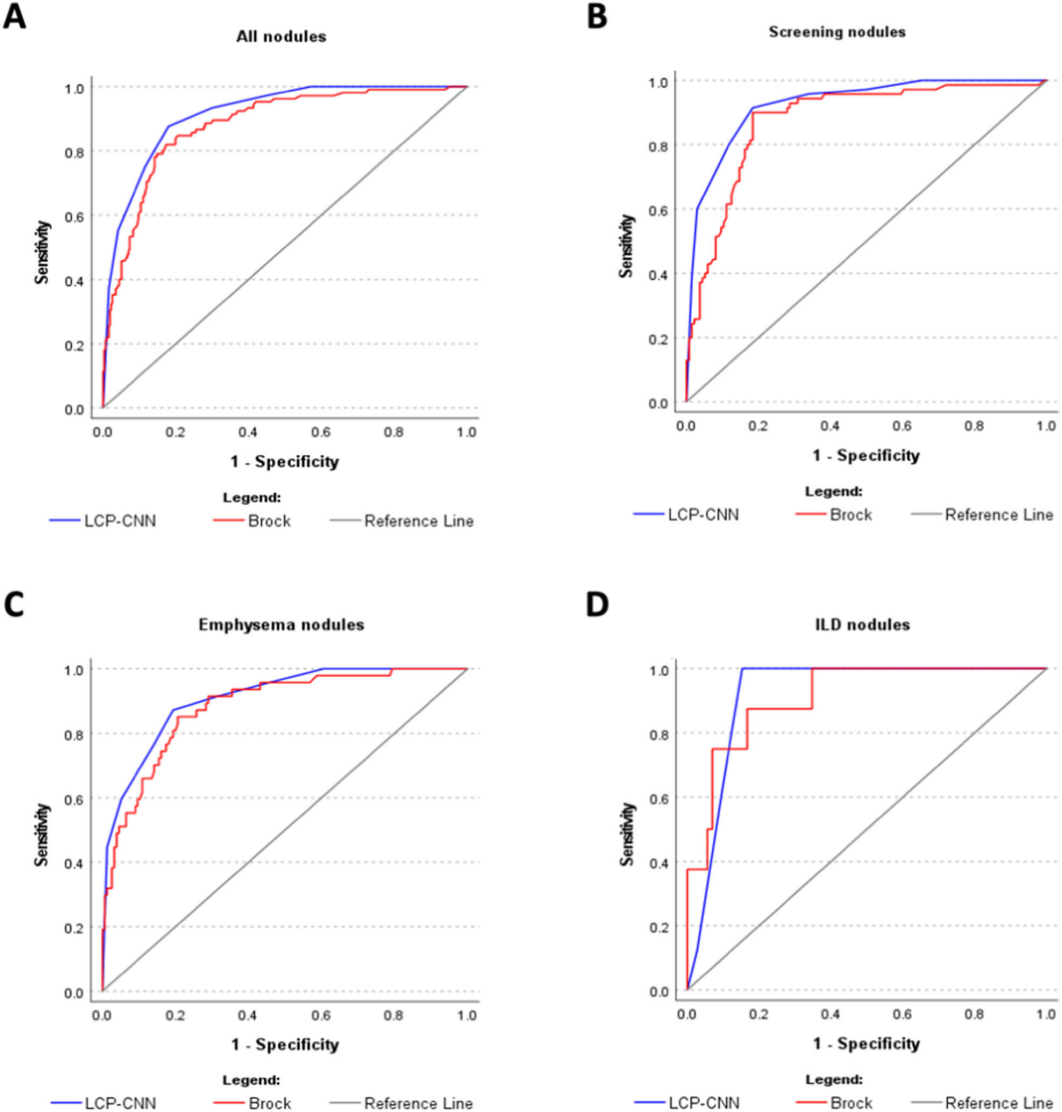
Comparison of the ROC for Brock model and LCP-CNN in the total cohort (**A**), in the screening cohort (**B**), in the emphysema cohort (**C**), and in the ILD cohort (**D**)

LCP-CNN achieved an AUC of 0.92 (95% CI: 0.89–0.94) in the total cohort, demonstrating significantly superior performance in classifying pulmonary nodules in terms of malignancy compared to the Brock model (AUC of 0.88, 95% CI: 0.84–0.92, *p* = 0.022) (Fig. 3A). These findings were replicated in the screening cohort, where LCP-CNN demonstrated an area under the curve (AUC) of 0.93 (95% CI: 0.89–0.96), which was significantly higher than the Brock model AUC of 0.88 (95% CI: 0.83–0.93, *p* = 0.016) (Fig. 3B).

In the cohort of patients with emphysema, LCP-CNN showed a similar AUC of 0.91 (95% CI: 0.86–0.95) compared to 0.88 (95% CI: 0.83–0.94) for the Brock model with the difference not being statistically significant (*p* = 0.232) (Fig. 3C). Equally, in the subcohort of patients diagnosed with ILD, LCP-CNN and Brock model did not show significant differences with an AUC of 0.92 (95% CI: 0.86–0.98) for LCP-CNN and 0.91 (95% CI: 0.82–1.00) for Brock model (*p* = 0.404) (Fig. 3D).

### LCP-CNN ROC performance shows no significant difference in patients with underlying pulmonary disease

LCP-CNN demonstrated comparable results for the classification of pulmonary nodules in all investigated subcohorts (Table 5), with an AUC ranging from 0.91 (95% CI: 0.86–0.95) in the emphysema cohort to 0.92 (95% CI: 0.89–0.94) in the total cohort and the ILD cohort (0.92, 95% CI: 0.86–0.98), and to 0.93 (95% CI: 0.89–0.96) in the screening cohort.

**Table 5:** Performance of the investigated models in terms of AUCs of the receiver operating statistics (ROC)

ROC		
Cohort/model	LCP-CNN [AUC (95% CI)]	Brock [AUC (95% CI)]
Total	0.92 (0.89–0.94)	0.88 (0.84–0.92)
Screening	0.93 (0.89–0.96)	0.88 (0.83–0.93)
Emphysema	0.91 (0.86–0.95)	0.88 (0.83–0.94)
ILD	0.92 (0.86–0.98)	0.91 (0.82–0.99)

## Discussion

Incidentally detected pulmonary nodules present an ongoing challenge as misclassification can lead to unnecessary procedures, costs, and uncertainty for patients, if misclassified as malignant, or delay of or no therapy, if misclassified as benign. Demand for reliable support systems for lung nodule classification therefore persists to this day. The increasing implementation of lung cancer screening is adding a different aspect to this problem with different risk characteristics of screening populations. It is essential to gain a comprehensive understanding of the potential and limitations of classification approaches.

The design of this study was based on a previous publication by Baldwin et al [16] in order to achieve comparable results and thus allow for the examination of possible reproducibility. In comparison, Baldwin et al analyzed 1397 pulmonary nodules collected at three sites (Leeds, Nottingham, and Oxford). The inclusion and exclusion criteria were similar to those used in this study, with minor differences (age of inclusion ≥ 18 years and nodule diameters of 5–15 mm compared to an age of inclusion ≥ 35 years and nodule diameters of 5–30 mm in our study). However, this study introduced further aspects. In addition to the comparison to the Brock model, the performance of the LCP-CNN AI-based classification tool was compared to Lung-RADS^®^. Furthermore, the respective performance of these models in different clinically derived patient cohorts, which differed by risk profile (screening cohort [25]) and/or underlying pulmonary disease (pulmonary emphysema [26] and ILD cohort [27, 28]), was analyzed.

In our study, LCP-CNN showed good results in the classification of pulmonary nodules into different risk categories with no significant differences when analyzing nodules in patients with pulmonary emphysema or fibrosis. It is noteworthy that even a screening cohort does not present a relevant challenge to the discrimination performance, despite the lack of specific adaptation of the LCP-CNN model for this purpose. Comparing results between the models we can show that LCP-CNN outperforms the Brock model in all cohorts in terms of AUC and sensitivity, except for the emphysema and ILD cohorts, while outperforming Lung-RADS^®^ in all investigated cohorts in terms of sensitivity. Specificity, however, was shown to be lower for LCP-CNN compared to the Brock model score and Lung-RADS^®^, pointing to a higher risk for overdiagnosis.

Overall results are comparable with the study by Baldwin et al with LCP-CNN reaching an AUC of 92% (95% CI: 89–94%) in our study compared to 90% (95% CI: 88–92%) for Baldwin et al These findings corroborate those of further studies, demonstrating AUC-values for LCP-CNN from 92% [13], 94% [29] to 95% [15].

In interpreting the results of this study, it is important to consider some limitations in the study design. The study was conducted retrospectively, only containing CT scans from a single center specializing in pulmonary diseases. This may potentially have led to a higher pretest probability for lung cancer, which could have influenced the classification performance of the LCP-CNN model [15]. Lung nodule assessment was primarily performed by only one reader with limited experience (a radiology resident with 1 year of experience), although under the supervision of an experienced chest radiologist as stated above. Furthermore, several assumptions were made that might result in a sampling error. Small nodules that remained stable over a 2-year period were considered benign by clinical convention, without undergoing a definite workup [30, 31]. To calculate the Brock model, a family history of lung cancer must be taken into account. Due to the retrospective nature of this study, for 136 patients this information was not available, and a negative family history was assumed, which may have implications for the performance of the model. This represents a relevant limitation of the Brock model, as it requires careful collection of patient data, which may not always be feasible in clinical practice.

In this analysis, Lung-RADS v.1.1 was used, as it was the current version, when CT examinations were conducted. The introduction of Lung-RADS^®^ 2022 might influence results, as DeSimone has recently shown the superiority of the new version with lower numbers in false-positive screening CT examinations [32]. This would likely serve to reinforce the high specificity of Lung-RADS^®^ demonstrated in this study.

The superior performance of the LCP-CNN score indicates its potential as a valuable tool in the risk stratification of pulmonary nodules, even in lung cancer screening programs. The deep learning-based approach offers efficiency and accuracy, addressing some of the limitations of traditional models, such as variations in manual measurements or the lack of patient data. Its use is straightforward and fast, reducing reading time compared to multiparametric statistical methods. Future research directions should involve further prospective validation studies in larger and more diverse cohorts. Consequently, this study offers further evidence of the potential to integrate deep learning-based decision support systems into pulmonary nodule classification workflows, irrespective of individual patient risk profile and underlying pulmonary disease.

## Abbreviations

AUC: Area under the curve
CI: Confidence interval
CT: Computed tomography
DLR: Diagnostic likelihood ratio
FDA: Food and Drug Administration
ILD: Interstitial lung disease
LCP-CNN: Lung cancer prediction-convolutional neural network
Lung-RADS^®^: Lung imaging reporting and data system
NPV: Negative predictive value
PPV: Positive predictive value
ROC: Receiver operating characteristics
SD: Standard deviation
